# Platelet lysate from whole blood-derived pooled platelet concentrates and apheresis-derived platelet concentrates for the isolation and expansion of human bone marrow mesenchymal stromal cells: production process, content and identification of active components

**DOI:** 10.3109/14653249.2012.655420

**Published:** 2012-05

**Authors:** Natalie Fekete, Mélanie Gadelorge, Daniel Fürst, Caroline Maurer, Julia Dausend, Sandrine Fleury-Cappellesso, Volker Mailänder, Ramin Lotfi, Anita Ignatius, Luc Sensebé, Philippe Bourin, Hubert Schrezenmeier, Markus Thomas Rojewski

**Affiliations:** 1 Institut für Transfusionsmedizin, Universität Ulm und Institut für Klinische Transfusionsmedizin und Immungenetik Ulm, DRK-Blutspendedienst Baden-Württemberg-Hessen, Germany; 2 Laboratoire de Thérapie Cellulaire, Etablissement Français du Sang-Pyrénées-Méditerranée Toulouse, France; 3 Institut für Unfallchirurgische Forschung und Biomechanik, Universitätsklinikum Ulm, Germany

**Keywords:** blood platelets, chemokines, cytokines, mesenchymal progenitor cells, mesenchymal stromal cells

## Abstract

**Background aims:**

The clinical use of human mesenchymal stromal cells (MSC) requires *ex vivo* expansion in media containing supplements such as fetal bovine serum or, alternatively, human platelet lysate (PL).

**Methods:**

Platelet concentrates were frozen, quarantine stored, thawed and sterile filtered to obtain PL. PL content and its effect on fibroblast-colony-forming unit (CFU-F) formation, MSC proliferation and large-scale expansion were studied.

**Results:**

PL contained high levels of basic fibroblast growth factor (bFGF), soluble CD40L (sCD40L), vascular cell adhesion molecule-1 (VCAM-1), intercellular adhesion molecule-1 (ICAM-1), platelet-derived growth factor AA (PDGF-AA), platelet-derived growth factor AB/BB (PDGF-AB/BB), chemokine (C-C) ligand 5 (CCL5; RANTES) transforming growth factor-β1 (TGF-β1) and chemokine (C-X-C) ligand 1/2/3 (GRO), with low batch-to-batch variability, and most were stable for up to 14 days. Inhibition of PDGF-BB and bFGF decreased MSC proliferation by about 20% and 50%, respectively. The strongest inhibition (about 75%) was observed with a combination of anti-bFGF + anti-PDGF-BB and anti-bFGF + anti-TGF-β1 + anti-PDGF-BB. Interestingly, various combinations of recombinant PDGF-BB, bFGF and TGF-β1 were not sufficient to promote cell proliferation. PL from whole blood-derived pooled platelet concentrates and apheresis platelet concentrates did not differ significantly in their growth-promoting activity on MSC.

**Conclusions:**

PL enhances MSC proliferation and can be regarded as a safe tool for MSC expansion for clinical purposes. \in particular, PDGF-BB and bFGF are essential components for the growth-promoting effect of PL, but are not sufficient for MSC proliferation.

## Introduction

The versatile application of mesenchymal stromal cells (MSC) for a diverse range of clinical indications has generated increasing interest in MSC as potential therapeutic agents in recent years. Research on MSC biology is progressing rapidly, as illustrated by the growing number of clinical trials using MSC registered on http://www.clinicaltrials.gov (accessed date: 31.07.2011). While MSC can be found in a variety of tissues and organs, most trials use MSC from either adipose tissue or bone marrow (BM). MSC comprise only 0.001–0.01% of bone mononuclear cells. Therefore extensive *ex vivo* expansion is required to obtain clinical doses (1) for cell therapy.

MSC comprise a heterogeneous population of plastic-adherent cells characterized by fibroblast-like morphology, the ability to form colonies and to differentiate along the mesodermal lineage into adipocytes, chrondrocytes and osteoblasts. Minimal criteria proposed by The International Society for Cellular Therapy (ISCT) also include cell-surface expression of CD73, CD90 and CD 105 as well as absence of CD11b or CD14, CD34, CD45, CD79α or CD 19 and HLA-DR (2). So far, there is no unique marker allowing for prospective isolation of MSC. Characterization of MSC has been hindered further by the observation that *in vitro* expansion changes the MSC phenotype and function, and leads to decreased clonogenicity as the cells enter replicative senescence after about 30 population doublings (3).

Translating research into clinical-scale manufacturing of MSC in accordance with good manufacturing practice (GMP) requires defined cell-culture conditions optimized to isolate and expand MSC *ex vivo* efficiently. The large-scale production of well-characterized media supplements is essential to maintain the cellular qualities required for the intended clinical application, while minimizing risks of adverse events.

As animal sera are ill-defined and pose a risk factor as a source of xenogenic antigens and possible transmitters of zoonotic infections, they are undesirable as medium supplements in cell therapy (4–6). Pooled human platelet lysate (PL) is a hemoderivate containing a plethora of growth-promoting factors and is being established as a safe and efficient MSC culture supplement for robust MSC cultivation. However, essential growth factors for optimal MSC culture have notbeen defined. Platelet-derived growth factor (PDGF), epidermal growth factor (EGF), insulin-like growth factor (IGF), basic fibroblast growth factor (bFGF), transforming growth factor-β1 (TGF-β1) and other factors have been subjected to investigation but could not replace serum supplements for efficient maintenance of MSC hallmark characteristics *ex vivo* (7–10).

Both PDGF and bFGF are highly potent mitogens for cells and act as regulators either by themselves or in combination with other factors. PDGF is a poly-peptide and consists of two disulfide-bonded amino acid chains that may form homo- or heterodimers that bind with different affinities to two different but structurally related cell-surface receptors. Human platelets contain all three isoforms, PDGF-AA, PDGF-AB and PDGF-BB (11,12). However, the biologic relevance and growth-promoting effects of any of these factors in human PL remain to be elucidated. bFGF is well known for stimulating cell proliferation of MSC (13) and is under investigation as an additional supplement to fetal bovine serum (FBS) in clinical trials (14). TGF-β1 is part of a large family of multifunctional cytokines, including TGF-β1, TGF-β2, TGF-β3 as well as activins and inhibins. AsTGF-P1 is secreted as a latent precursor molecule mainly found in association with the matrix, it requires activation to allow the TGF-β1 protein to bind to its receptors (15). TGF-β1 is a pleiotropic cytokine involved in cellular proliferation, differentiation and migration, recruiting neutrophils, macrophages and fibroblasts to the site of inflammation (16).

In this study, we developed a GMP-grade protocol for large-scale expansion of human MSC from clinical-grade platelet concentrates (PC). We compared PL prepared from different types of PC (pooled whole blood-derived PC versus apheresis PC). We characterized its major components, its batch-to-batch variability and stability. By carrying out neutralization experiments, we identified the factors that are essential for the stimulating activity on MSC proliferation.

## Methods

### Production of PL front whole blood-derived pooled PC

Blood donors were tested according to guidelines for the preparation of blood and blood components and the use of blood products (*Hemotherapy Guidelines*) according to §§ 12 and 18 of the German transfusion law (17).

**Figure 1:**
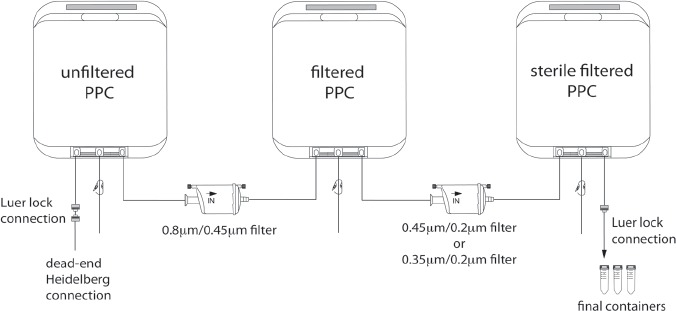
Scheme of the filtration process for the production of PL-PPC. Whole blood-derived PL concentrates passing quarantine were thawed, centrifuged, pooled in a 5-L bag and pre-filtered through a filter with pore sizes of 0.80/0.45 μm and sterile filtered into a second 5-L bag. In a second step, the final filtration using a filter with pore sizes of 0.45/0.35 μm was performed into a third 5-L bag and filled into containers of suitable sizes.

Whole blood from healthy volunteer donors was stored for 3–22 h prior to centrifugation to obtain buffy coats. Four buffy coats were pooled in an additive solution (Viaflex-Beutel® 200 mL T-Sol, Baxter, Unterschleißheim, Germany; or Macoflex N 200 mL Thrombozytenkonservierungslösung, MacoPharma, Langen, Germany) (17). Pooled PC (PPC) were stored at + 22°C ± 2°C for no longer than 5 days, irradiated with 30 Gy (Biobeam8000; GammaService, Leipzig, Germany) and frozen at −30°C. Quarantine storage was performed at −30°C to −45°C for up to 18 months. PPC were released for clinical-grade PL production only if all four donors contributing to a PPC had tested negative again after an interval of at least 4 months for the following infectious disease markers: human immunodeficiency virus (HIV) [by polymerase chain reaction (PCR) and serologic testing]; hepatitis C virus (HCV) (by PCR and serologic testing), hepatitis B (HBV) (by PCR, hepatitis B surface antigen (HBsAg) and anti-hepatitis B core protein (anti-HBc)), hepatitis A (HAV) (by PCR), parvovirus B19 (by PCR < 10^5^ U/mL) and *Treponema pallidum* (serologic testing). About 30% of frozen PC passed quarantine within 2 years. PPC passing quarantine were thawed at 37°C using a Plasmatherm device (Labor Technik Barkey, Leopoldshohe, Germany) for 13 min and stored at 37°C for a further 2 h. Up to three PPC were transferred into one 500-mL Compoflex bag (Compoflex Einfach Blutbeutel 500 mL; Fresenius Kabi, Bad Homburg, Germany) by sterile welding, using a Terumo TSCDII (Terumo, Leuven, Belgium), and stored overnight at 4°C. PPL in Compoflex bags were centrifuged at 20°C for 10 min at 4000 r.p.m. using a Hettich Roto Silenta 63 RS centrifuge (Hettich, Tuttlingen, Germany). The supernatant was transferred and pooled in 2-L bags (Transfer Pack Containers 2000 mL; Baxter, Unterschleissheim, Germany) by using a mechanical press. These 2-L bags were further processed in GMP class B clean rooms. Each 2-L bag of one batch was welded consecutively to the dead end of a Heidelberger extension (B. Braun, Melsungen, Germany) and connected via a Luer-Lock connection to a 5-L bag (STD FLEXBOY 5L; Sar-torius Stedim Biotec, Melsungen, Germany). A filter system was set up in a GMP class A biologic safety cabinet, taking care to maintain the correct liquid flow direction in the filters (Figure 1). For the removal of large particles, a first filtration step was performed using a 0.8/0.45-μM filter with a surface of 0.05–0.6 m^2^ (Sartopore 2 Membran-filtercapsule; Sartorius Stedim Biotec). A second sterile filtration step was performed using a 0.45/ 0.2-μM or 0.35/0.2-μM filter with a surface of 0.05-0.6 m^2^ (Sartopore 2 Membranfiltercapsule; Sartorius Stedim Biotec). All filtration steps were performed using a peristaltic pump (620 S/R; Wat-sonMarlow, Rommerskirchen, Germany). After the two filtration steps, the PL from whole blood-derived PPC (PL-PPC) was filled into 50-mL tubes (Greiner Bio-one GmbH, Frickenhausen, Germany) or 100-mL, 500-mL or 1000-mL bottles (Greiner Bio-one GmbH). The final product was tested for sterility by BacT/ALERT (bioMérieux, SA, Marcy-l'Etoile, France). The endotoxin concentration was below 1 endotoxin unit (EU)/mL, as measured by a Limulus assay according to the European Pharmacopoeia (18). The filtration steps of PL did not change the growth-promoting effect on the MSC (data not shown). The final product had a shelf-life of 2 years and was stored at −80°C to −30°C until use.

### Production of PL from apheresis PC

Blood donors were tested according to the French regulations for the preparation of blood components. They were negative for HIV, HBV, HCV, human T-lymphotropic virus type 1 (HTLV-1) (PCR and serologic testing) and syphilis (serologic testing). PC were obtained from apheresis (APC) and the platelets were kept in the donor plasma. The concentrates were rejected if the platelet concentration was below 10^9^ platelets/mL. We did not pool the PC from different donors. After sterility testing (BacT/ALERT; bioMerieux SA), the PC were kept at + 22°C ± 2°C for 5 days under constant agitation. They were processed afterwards when they were no longer valid for transfusion. They were then frozen in a cryopreservation bag (MacoPharma, Tourcoing, France) at −80°C. For further processing, a system from MacoPharma was used that consisted of three components: a freezing and centrifugation bag, a filtration kit and a distribution kit. After a quarantine of 4 months, if the donor was still negative for the above-mentioned markers, the PC underwent two cycles of thawing at + 37°C and freezing at −80°C, and were subsequently thawed at + 4°C for at least 12 h and then centrifuged for 30 min. The supernatant was filtered by gravity using a 0.65-μM filter connected to a bag (MacoPharma) for no longer than 30 min. The bag containing filtered PL was recovered by sealing and connected to a 9-bag distribution kit provided by MacoPharma. The entire procedure was done in closed systems. The PL from apheresis PC (PL-APC) were aliquoted in either 30-mL bags (MacoPharma) or tubes.

### Source of MSC

All MSC used for the following experiments were primary MSC, collected from BM aspirations (iliac crests) from volunteer healthy donors. MSC were used at passages 0–2. Collection of this material had been approved by the ethical committee of the University of Ulm (Ulm, Germany) and the informed consent of patients and healthy donors had been obtained.

### Proliferation assay

Cell proliferation was assessed using the CyQUANT® cell proliferation assay kit (Invitrogen, Grand Island NY, USA) according to manufacturer's instructions after 7 days of culture with the indicated conditions. Fluorescence was measured in relative fluorescence units (RFU) using a POLARstar Omega plate reader (BMG Labtech, Ortenberg, Germany) and the background signal of the lysis buffer was subtracted.

### Addition of recombinant human proteins and neutralizing human PDGF-BB

Recombinant human PDGF-BB (BioLegend, San Diego, CA, USA) was used at a final concentration of 20 ng/mL to mimic the concentration present in 10% PL. Recombinant human bFGF 146 aa (R&D Systems Inc., Minneapolis, MN, USA; 20 μg/mL) and recombinant human TGF-β1 (R&D Systems Inc.; 100 ng/mL) were used either by themselves or in combination as supplementing factors in addition to alpha-minimal essential medium (MEM) to stimulate MSC proliferation.

### Neutralizing assay

The role of PDGF-BB, bFGF and TGF-β1 as components of PL on MSC proliferation was assessed by the addition of neutralizing antibodies to alpha-MEM medium supplemented with 10% PL. Goat anti-human PDGF-BB antibody (R&D Systems Inc.; 10 μg/mL), goat anti-human bFGF antibody (R&D Systems Inc.; 120 μg/mL) and mouse anti-human TGF-β1 (R&D Systems Inc.; 60 ng/mL) were used according to the manufacturer's instructions. The goat anti-human antibody was produced with *Escherichia* co/z-derived recombinant human bFGF as immunogen. Goat anti-human IgG (R&D Systems Inc.; 100 μg/mL) was used as a control.

Mouse anti-human vascular endothelial growth factor (VEGF) antibody (1 μg/mL), mouse anti-human PDGF-AA antibody (5 μg/mL), mouse anti-human chemokine ligand 5 (CCL5; RANTES) antibody (15 μg/mL), mouse anti-human chemokine (C-X-C) ligand (CXCL) 1/2/3 pan-specific antibody (5 μg/mL), mouse anti-human CD40 ligand antibody (5 μg/mL), mouse anti-human chemokine (C-C) ligand (CCL3)/macrophage inflammatory protein (MIP)-lα antibody (1 μg/mL), mouse anti-human CCL4/MIP-1β antibody (1 μg/mL), mouse anti-human vascular cell adhesion molecule-1 (VCAM-l)/CD106 antibody (5 μg/mL) and mouse anti-human intercellular adhesion molecule-1 (ICAM-1)/CD54 antibody were all purchased from R&D Systems Inc. and used according to the manufacturer's instructions. Cell proliferation in the respective culture conditions was quantified following 7 days of culture.

### Modified fibroblast-colony-forming unit assay

Fibroblast-colony-forming units (CFU-F) were set up by seeding non-manipulated fresh BM (less than 1 h between the collection and assay for comparison of PL-PPC and PL-APC) at a density of 12 000 BM mononuclear cells (MNC)/cm^2^ in alpha-MEM (Lonza, Basel, Switzerland) supplemented with either 10% PL of different batches of both PL-PPC and PL-APC or 20% FBS (Invitrogen, Darmstadt, Germany). After 72 h non-adherent cells were removed by washing with phosphate-buffered saline (PBS) without Ca^2+^/Mg^2+^ (Lonza). Medium exchange was performed twice per week. Colonies consisting of more than 5 cells were counted after 7 days of culture to determine clonogenicity using different PL batches. This modification allowed for a faster detection and also included small, slow-proliferating colonies. Scoring errors because of confluence were avoided by using this procedure. The doubling time was determined on the basis of CFU-F after 10 days using the following equation: doubling time = 1n(2) × culture time (h)/1n[total cell number/(CFU-F/BM MNC) × cell number seeded]. Cell counting was performed using trypan blue to discriminate dead cells.

### Large-scale expansion

Unprocessed BM was seeded at a density of 12 000 cells/cm^2^ in 750 mL alpha-MEM (Lonza, Basel, Switzerland) supplemented with 10% PL per 5-chamber stack (Corning, Amsterdam, the Netherlands). After 72 h, non-adherent cells were removed by washing with PBS without Ca^2+^/Mg^2+^ (Lonza). A partial medium exchange was performed twice a week by replacing 300 mL. The cells were harvested after 10 days, using TrypZEAN (Lonza). Cell counting was performed after trypan blue exclusion of dead cells.

For comparative analysis of PL-APC and PL-PPC, frozen PC from Toulouse, France, were shipped to Ulm, Germany. Small- and large-scale MSC expansions using PL-APC and PL-PPC were performed in Ulm.

### Flow cytometric characterization and differentiation assays

Antibodies used for the characterization of MSC included CD3 (SK7), CD34 (8G12), CD45 (2D1), CD73 (AD2), CD90 (5E10), HLA-DR, DP, DQ (Tü39), HLA-ABC (G46-2.6) (Becton Dickinson, Heidelberg, Germany) and CD 105 (SN6) (AbD Serotec, Düsseldorf, Germany). 10^6^ cells were incubated with antibodies for 15 min, washed with PBS (Lonza, Switzerland) and the relative fluorescence intensity of cells was acquired using a FACScan with CellQuest Software (BD Immunocytometry Systems, Heidelberg, Germany). For differentiation assays, 2.75 × 10^3^–10^4^ cells/cm^2^ were seeded and differentiation was induced according to the manufacturers’ instructions (adipogenic differentiation medium from Lonza; chondrogenic and osteogenic differentiation media from Miltenyi, Bergisch-Gladbach, Germany). After differentiation, cells were fixed in 4% paraformaldehyde and osteogenic differentiation was detected showing alkaline phosphatase activity. Adipogenic differentiation was monitored by staining with a saturated Oil Red O solution (counterstaining with Meyer's hematoxylin). Chondrogenic differentiation was performed by Methylene Blue staining. All reagents for staining were purchased from Sigma (Schnelldorf, Germany) except for Methylene Blue (Merck, Darmstadt, Germany).

### Cytokine analysis

**Figure 3:**
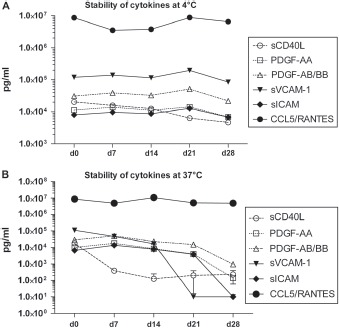
Stability of cytokines. (A) Human cytokine analysis of one PL-PPC batch stored at +4°C. Starting just after production, the concentration of six highly present soluble factors was evaluated by multiplex cytokine dosage every week for 4 weeks. (B) Human cytokine analysis of one PL-PPC batch incubated at + 37°C. Starting just after production, the concentration of six highly present soluble factors was evaluated by multiplex cytokine dosage every week for 4 weeks.

Custom-designed MILLIPLEX® human cytokine/chemokine 96-well plate assays (catalog number MPXCYTO-60K, HNDG3–36K; Millipore Corporation, Billerica, MA, USA) and a TGF-β Single Plex Kit (catalog number TGFB-64K-01) were used for the simultaneous quantification of human cytok-ines and chemokines of cell culture supernatants and human PL preparations as per the manufacturer's specifications. For stability analysis of PL, one PL batch was thawed and aliquots were then incubated at either 4°C or 37°C for up to 28 days. At each time-point of interest, the aliquot was then stored at −80°C for simultaneous measurement via the MILLIPLEX cytokine assay. As freezing/thawing cycles affect the absolute concentrations of cytokines (data not shown), samples were processed in such a way that they did not differ in number of freezing/thawing cycles. This affected the absolute concentrations but allowed assessment of stability over time, as presented in Figure 3.

The TGF-β1 concentration in PL was also analyzed quantitatively, using a Quantikine enzyme-linked immunosorbent assay (ELISA) kit (DB100; R&D Diagnostics, Wiesbaden, Germany) following the manufacturer's protocol. A dilution series of TGF-β1 standard was prepared; 100 μL PL were activated with 100 μL 2.5 N acetic acid/10 M urea, incubated at room temperature for 10 min, and neutralized with the addition of 100 μL 2.7 N NaOH/1 M HEPES. Standard and samples were added to a 96-well microtiter plates coated with TGF-β1 receptor II. By adding a polyclonal antibody against TGF-P1 conjugated to horseradish peroxidase, binding was visualized via a chromogen reaction. Three dilutions were measured, each in duplicate. The results are expressed as mean ± standard deviation (SD). The cytokine analysis of PL-APC and PL-PPC was performed in Ulm, Germany.

### Statistics

Data are expressed as mean ± SD. The statistical significance of the effect of inhibition of bFGF, TGF-β and/or PDGF-BB, as well as the addition of recombinant PDGF-BB to the cell culture medium on MSC proliferation, was assessed using a non-parametric two-tailed Wilcoxon signed rank test. Statistical analysis was performed with GraphPad Prism software for Windows, Version 5.02 (Graph-Pad Software Inc., La Jolla, CA, USA).

## Results

### Yield

Starting with 6105 ±205 mLPPC, a yield of 84 ± 3% was achieved, resulting in 5132 ±214 mL PL-PPC after the production process, as tested with a total of 13 batches.

### Major components of PL-PPC

**Figure 2:**
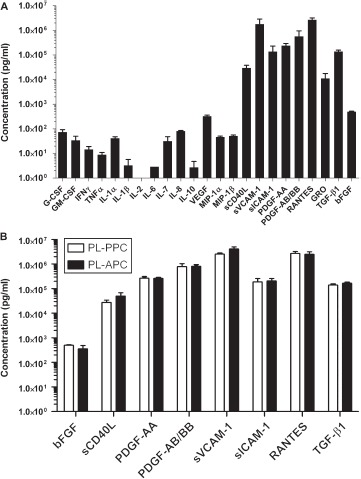
Multiplex analysis of human PL. (A) Cytokine concentrations of different PL-PPC preparations in pg/mL. Number of analyzed batches: *n* = 3 for G-CSF, GM-CSF, INF-γ, TNF-α, IL-lα, IL-1β, IL-2, IL-6, IL-7, IL-8, IL-10, VEGF, MIP-lα, MIP-1β, GRO (CXCLl/2/3), TGF-β1 and bFGF; and *n* = 9 for sCD40L, sVCAM-1, sICAM-1, PDGF-AA, PDGF-AB/BB and RANTES. (B) Comparing cytokine content of PL-PPC and PL-APC. Number of analyzed batches: *n* = 3 forTGF-β1 and bFGF in PL-APC and PL-PPC; *n* = 4 for sCD40L, sVCAM-1, sICAM-1, PDGF-AA, PDGF-AB/BB and RANTES in PL-APC; and *n =* 6 for these cytokines in PL-PPC. Open bars depict PL-PPC and filled bars PL-APC.

The average protein content of PL-PPC (*n* = 13) was 26 ± 5 g/L, with 62 ±1% albumin. To investigate the human cytokine content of PL-PPC, we used a custom-designed 96-well plate Multiplex assay. The analysis of three different PL preparations revealed the following levels: granulocyte-colony-stimulating factor (G-CSF) (74 ± 19 pg/mL), granulocyte-macrophage (GM)-CSF (34 ±16 pg/mL), interferon (IFN)-γ (14 ±4 pg/mL), tumor necrosis factor (TNF)-α (8 ± 2 pg/mL), interleukin (IL)-1α (41 ±6 pg/mL), IL-1β (3 ±2 pg/mL), IL-2 (0 ± 0 pg/mL), IL-6 (3 ± 0 pg/mL), IL-7 (32 ± 16 pg/mL), IL-8 (80 ±6 pg/mL), IL-10 (3 ± 2 pg/mL), VEGF (325 ± 34 pg/mL), MIP-1α (47 ± 4 pg/mL), MIP-10 (51 ±5 pg/mL), sCD40L (29 738 ±8361 pg/mL), soluble vascular cell adhesion protein 1 (sVCAM-1) (1 789 695 ± 1 108 320 pg/mL), soluble intercellular adhesion molecule 1 (sICAM-1) (137 300 ± 93 670 pg/mL), bFGF (495 ± 27 pg/mL) and TGF-β1 (139 029 ± 18 854 pg/mL). The highest concentrations were found for PDGF-AA (239 412 ± 53 690pg/mL), PDGF-AB/BB 571 730 ± 381 036pg/mL) RANTES/CCL5 (2 705 600 ± 496 076 pg/mL) and chemokine (C-X-C) ligand 1/2/3 (GRO) (= CXCL1/2/3) (11 126 ±6480 pg/mL). A large amount of soluble adhesion molecules was also detected: sCD40L (29 738 ±8361 pg/mL), sVCAM-1 (1 789 695 ± 1 108 320 pg/mL) and sICAM-1 (137 300 ± 93 670 pg/mL) (Figure 2A).

To determine whether the preparation method or the platelet source may result in a distinct cytokine profile of PL, we then compared four PL lots from PPC with those derived from APC using the Multiplex assay. The results depicted in Figure 2B show no significant differences in cytokine content of PL derived from PPC or APC.

### Stability of PL

Having profiled the absolute concentrations of these 22 PL components, we then decided to investigate the stability of six of these factors that were present at concentrations about or higher than 10 ng/mL, as a temporal decline of these factors would be easily detectable. Although concentration by itself is not necessarily indicative of biologic significance, these components are known to act as growth and adhesion factors, which are likely candidates for the promotion of MSC proliferation.

Cytokine analysis of different PL-PPC and PL-APC revealed comparable amounts of PDGF-AB, PDGF-BB, sICAM-1, sVCAM-1, RANTES/CCL5, bFGF, TGF-β1 and sCD40L after thawing. It may be assumed that a certain threshold of cytokines and growth factors is necessary, and even indispensable, for cell growth. In order to establish for how long unfrozen PL can be used, we analyzed cytokine levels of PL-PPC that were stored at + 4°C or + 37°C after thawing, respectively.

As depicted in Figure 3A, storage of PL at + 4°C for 28 days resulted in declining levels of sCD40L from day 0 and stable levels of PDGF-BB, sICAM-1, sVCAM-1 and CCL5. When PL was incubated with standard MSC cell culture conditions (+ 37°C, 5% CO_2_), the cytokine profile showed stable levels of RANTES and progressively declining levels of all the other factors tested. However, PDGF-BB levels seemed to be stable up to at least 7 days.

### Growth-promoting effect of PL-PPC and PL-APC

**Figure 4:**
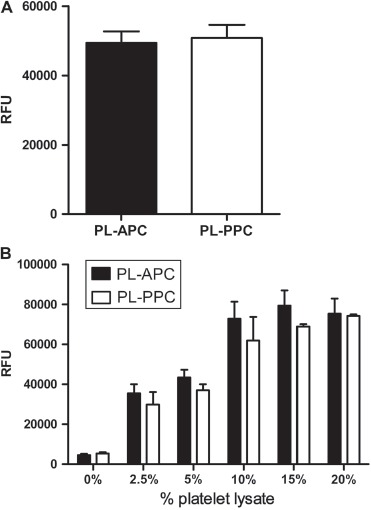
Proliferation capacity of PL-PPC and PL-APC. (A) Comparison of MSC proliferation in 10% PL-APC (*n* = 4) and 10% PL-PPC (*n* = 6). (B) MSC proliferation assays were performed by comparing different batches of PL-PPC (*n* = 6) and PL-APC (*n* = 2) as a culture supplement at concentrations ranging from 0 to 20%. Results are expressed as mean and SD values of three independent experiments. RFU, relative fluorescence units.

PL batches that were prepared from PPC either on day 2 or day 6 after donation did not differ in their growth-promoting effects on MSC. We also compared one versus two freezing cycles to lyse the platelets, and there was no significant impact of the number of freezing cycles on the stimulatory activity of PL on MSC proliferation (data not shown). No significant differences in growth-promoting activity could be observed when MSC were cultivated with 10% of either PL-PPC and PL-APC (Figure 4A). To test for different minimal thresholds of the cell-promoting efficacy of both supplements, the medium was supplemented with increasing concentrations of PL. A dose-dependent proliferation of MSC could be observed when pre-established MSC were cultured in alpha-MEM medium supplemented with heparin and 0–20% PL derived from either PPC or APC (Figure 4B). No significant difference between PL-PPC and PL-APC was observed. The optimal concentration of PL was determined as 10% as 15% and 20% of PL did not result in a significant increase in cell proliferation.

We then compared the effect of PL from either PPC or APC, at the optimal concentration, on the proliferation of primary MSC from fresh BM aspirates. In order to exclude inter-donor variability, the comparisons were performed with BM of the same donor. Cultures with either PL-PPC or PL-APC did not differ significantly in number of CFU-F, doubling time of MSC and number of MSC harvested per 10^6^ BM MNC seeded (Table I).

**Table I:** Analysis of growth-promoting effects of PL-PPC and PL-APC. Six batches of PL-PPC and three batches of PL-APC were used to compare MSC proliferation and clonogenicity parameters in T-25 culture flasks according to a GMP-grade protocol.

Parameter assessed	PL-PPC (*n* = 6)	PL-APC (*n* = 3)	*P*
CFU-F/10^6^ BM MNC seeded	302 ± 32	296 ± 30	0.503
MSC harvested/10^6^ BM MNC seeded	4.4 × 10^6^ ± 0.3 × 10^6^	5.2 × 10^6^ ± 1.4 × 10^6^	0.699
MSC harvested/ μL BM seeded	3.8 × 10^4^ ± 0.3 × 10^4^	4.5 × 10^4^ ± 1.2 × 10^4^	0.362
Doubling time (h)	22.5 ± 0.1	22.2 ± 0.7	0.371

*P*, *P*-value of Student's *t*-test.

### Growth-promoting effect of PL-PPC compared with FBS

Table II summarizes the results using alpha-MEM, supplemented with 20% FBS and medium completely free of animal origin (alpha-MEM supplemented with 10% PL-PPC) for the isolation and expansion of MSC. No significant difference in doubling time, the formation of CFU-F and the number of MSC harvested per 10^6^ BM MNC was observed.

**Table II:** Comparison of growth-promoting effects of PL-PPC and FBS in T-25 culture flasks.

Parameter assessed	PL-PPC (*n* = 6)	FBS (*n* = 6)	*P*
CFU-F/10^6^ BM MNC seeded	132 ± 133	156 ± 159	0.778
MSC harvested/10^6^ BM MNC seeded	5.6 × 10^5^ ± 6.3 × 10^5^	4.3 × 10^5^ ± 3.9 × 10^5^	0.075
MSC harvested/ μL BM seeded	5.5 × 10^3^ ± 6.4 × 10^3^	3.9 × 10^3^ ± 4.3 × 10^3^	0.667
Doubling time (h)	26.6 ± 2.9	23.8 ± 1.7	0.629

*P*, *P*-value of Student's *t*-test.

### Large-scale isolation and expansion with PL

To test the suitability and feasibility of both PL-PPC and PL-APC in large cell-expansion systems, a single-step large-scale isolation and expansion assay was performed using BM from one donor (Table III). Both supplements were suitable for large-scale isolation and expansion of MSC in a five-chamber stack system, leading to MSC doubling times below 20 h. Seeding 3.8 × 10^7^ BM MNC per five-chamber stack resulted in 3.2 × 10^7^ and 6.7 × 10^7^ MSC after 10 days of culture for PL-PPC and PL-APC, respectively.

**Table III:** Comparison of growth-promoting effects of PL-PPC and PL-APC in a 5-chamber stack system performed according to a GMP-grade standardized protocol.

Parameter assessed	PL-PPC	PL-APC
CFU-F/10^6^ BM MNC seeded	150	140
MSC harvested/10^6^ BM MNC seeded	0.9 × 10^6^	1.7 × 10^6^
MSC harvested/ μL BM seeded	0.8 × 10^6^	1.2 × 10^6^
Doubling time (h)	19.2	17.4

### Characterization of large-scale expanded MSC using PL

Flow cytometry analysis of large-scale expanded MSC (see Supplementary Figure 1) using markers to detect residues of BM cells (CD3, CD34, CD45 and HLA-DP, DQ, DR) as well as standard characterization markers for MSC (CD90, CD73, CD105 and HLA-ABC) defined pure MSC populations, with more than 95% of the cells being positive for CD73, CD90 or CD 105. Less than 1% of the expanded cells were positive for CD3, CD34 or CD45. MSC expanded in medium containing PL-PPC and PL-APC both showed adipogenic, chondrogenic and osteogenic differentiation (see Supplementary Figure 2).

### Influence of PDGF-BB on MSC proliferation

As we could show that PL contained high concentrations of PDGF-AA/AB, PDGF-BB, RANTES and TGF-β1 as well as noticeable amounts of VEGF, GRO, MlP-lα, MIP-1β, VCAM-1, ICAM-1, bFGF and sCD40L, we were interested in identifying the factors responsible for the growth-promoting activity of PL and evaluating the effects of these factors in MSC culture. Therefore, we inhibited these factors in PL using neutralizing antibodies and assessed MSC proliferation after 7 days in culture using a fluorescence-based quantification method.

**Figure 5:**
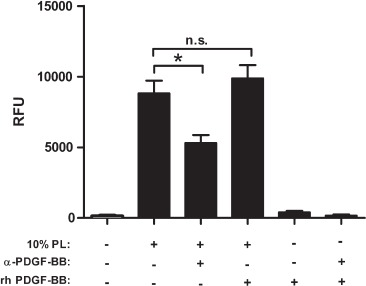
Inhibition of PDGF-BB by a monoclonal antibody significantly reduces proliferation of MSC in medium supplemented with 10% PL (*P* < 0.001). Addition of recombinant human PDGF-BB (20 ng/mL) did not enhance proliferation compared with 10% PL alone (*P*=0.47). Shown are mean and SD values of quadruplicate wells of one representative experiment. Each experiment was repeated at least twice. RFU, relative fluorescence units; rh, recombinant human; a, and; n.s., not significant.

**Figure 6:**
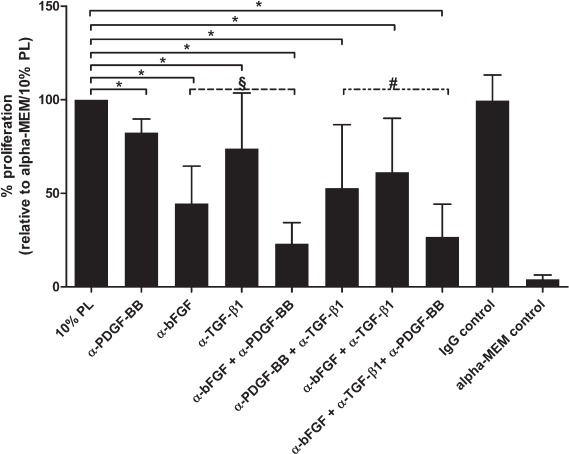
Inhibition of PL components bFGF, PDGF-BB and TGF-β1 by neutralizing antibodies as single factors or in combination significantly reduces MSC proliferation. Data are presented as mean and SD values of quadruplicate wells of three independent experiments. Statistical significance of the effect of inhibition of bFGF, TGF-β1 and/or PDGF-BB on MSC proliferation: *P<0.05 compared with MSC cultivated in alpha-MEM/10% PL; *P* = 0.039 compared with anti-PDGF-BB+ anti-TGF-β1; §*P* = 0.039 compared with anti-PDGF-BB. α, anti.

No effect on MSC proliferation could be detected after inhibition of any of the single factors PDGF-AA, RANTES, VEGF, GRO, MIP-1α, MIP-1β, VCAM-1, ICAM-1 and sCD40L (data not shown). Thus, despite the presence of a high concentration of some components, for example CD40L and RANTES, they did not seem to contribute to the growth-promoting effect of PL as single factors. Only a combination of sCD40L, VEGF and GRO, or RANTES, VEGF and GRO, or sCD40L, MIP-lα and MIP-1β reduced MSC proliferation by merely about 22%, 10% and 16%, respectively (data not shown). However, inhibition of PDGF-BB in medium supplemented with 10% PL significantly decreased MSC proliferation (Figures 5 and 6). Interestingly, recombinant human PDGF-BB added to alpha-MEM in concentrations equivalent to the amount present in PL did not stimulate MSC proliferation. The addition of recombinant PDGF-BB to 10% PL-PPC did not significantly enhance MSC proliferation compared with 10% PL-PPC (Figure 5).

### Inhibition of PL-induced MSC proliferation

MSC proliferation could be decreased significantly by inhibition of PDGF-BB, bFGF, TGF-β1 or a combination of these factors (Figure 6). Compared with MSC cultivated in medium supplemented with 10% PL, inhibition of bFGF by itself had the strongest effect as a single factor. Further, inhibition of both PDGF-BB and bFGF showed significant inhibition that was higher than the inhibition of either PDGF-BB or bFGF by itself. This effect could not be seen for the inhibition of either combination of PDGF-BB and TGF-β or bFGF and TGF-β. Finally, while combined inhibition of PDGF-BB, TGF-β1 and bFGF could significantly increase inhibition compared with the double inhibitory combination of PDGF-BB and TGF-β1, this triple combination of inhibitors could not further decrease the proliferation of MS C compared with the combination of anti-PDGF-BB and anti-bFGF. In conclusion, the strongest decreasing effect on MSC proliferation in 10% PL medium could be achieved by inhibition of bFGF in combination with anti-PDGF-BB, and there was no additional increase in inhibition when using a combination of anti-bFGF, anti-PDGF-BB and anti-TGF-β1. Based on these results we then tried to stimulate MSC proliferation with a cocktail of recombinant human (rh) PDGF-BB, rhbFGF and rhTGF-β1. However, neither alone nor in combination were these factors able to stimulate MSC proliferation comparably to 10% PL (see Supplementary Figure 3).

## Discussion

*Ex vivo* expansion of MSC requires the addition of supplements to the basal culture medium. FBS-based expansion protocols have been used in most of the early clinical trials (19,20). However, there are increasing safety concerns regarding use of FBS in clinical-grade expansion protocols (21,22). MSC can internalize protein components of FBS and elicit an immune response (23,24). This immune response might affect the clinical efficacy of MSC (23–25). FBS also contains non-human sialic acid Neu5Gc, against which many humans have circulating antibodies (26). It has been demonstrated that exposure to antibodies specific for Neu5Gc results in killing of human embryonic stem cells *in vivo.*

FBS can transmit infectious agents, for example transmittable spongiform encephalopathy (TSE) (27,28), and therefore the European Medicine Agency (EMA) recommends a preference for materials from a non-animal origin or non-TSE-relevant animal species (6). Thus non-animal alternatives are warranted, either serum-free media (29) or human supplements such as serum or PL (30–39).

However, human alternatives also have safety aspect considerations. There is the potential risk of transmission of infectious disorders if PL are prepared from allogeneic donors. The use of autologous platelets would limit the amount of PL and not be sufficient for large-scale expansion. Also, the preparation of PL in small amounts can result in considerable batch-to-batch variability. Our approach was to minimize the variability and produce a large amount of PL that was sufficient for several clinical-scale expansions. To this end, we used PC that were prepared and released for therapeutic platelet transfusions. The donors had to fulfill stringent blood donor eligibility criteria, including negative results for infectious disease markers (HIV, HBC, HCV, HAV, parvovirus B19 and *Treponema pallidum).* With this approach, including nucleic acid amplification technique, testing the residual risk of viral transmissions has been estimated at 1:360 000 for HBC, 1:4.3 million for HIV and 1:10 88 for HCV (40). The production process of PL in fact includes a freezing process. We took advantage of this to extend the cryopreservation of individual PC and to release the PC for pooling and further processing of clinical-grade PL, although only if the donors had further tested negative for all infectious disease markers on the occasion of a subsequent donation. Thus, for production of clinical-grade PL we used a procedure analogous to quarantine of therapeutic fresh frozen plasma. This further reduces the residual risk of a window period donation (time frame of donation) (41).

Thus we have designed a very safe human medium supplement. One might consider the use of autologous PL. However, this solution encounters at least two major hurdles, (i) In autologous treatment, patients are generally sick and it may be difficult to harvest PC safely and to harvest BM. (ii) In allogeneic settings, the amount of cells that must be produced, and thus the volume of required culture medium, surpasses the possibility of using one PC from the BM donor.

The efficacy of PL in terms of MSC proliferation did not differ regardless of whether it was prepared from PPC on day 2 or day 6 after donation. Thus it is possible to use PPC at the end of their shelf-life (in Germany, currently 4 days starting at midnight of the day of donation) if they are not required for transfusion. This approach allows the use of PPC either for therapeutic platelet transfusion or as starting material for preparation of PL. We can use the donor's gift optimally and avoid competition between PPC production versus PL production. In many institutions both PPC derived from whole blood donations and single donor apheresis PC are available (42). We therefore compared the two sources and could not detect substantial differences in terms of cytokine content or stimulation of MSC proliferation.

Comparing the growth-promoting effects of six batches of PL-PPC and three batches of PL-APC on MSC proliferation and clonogenicity in a ‘small-scale', approach we could not find any significant differences regarding the doubling time, CFU-F content or MSC/μL BM seeded. We also performed a large-scale MSC isolation and expansion run according to a GMP-grade protocol using PL-PPC and PL-APC, and could confirm the feasibility and efficacy of these two supplements in a clinical setting. While we observed some variability in the number of MSC harvested/μL BM seeded, the CFU-F/10^6^ BM MNC seeded and the doubling time were equivalent in both systems.

As already described by others (31,35,36), we confirmed that PL contains large amounts of PDGF-AB, PDGF-AB/BB and TGF-β1, albeit the concentrations reported by the various groups differ substantially. In addition, we demonstrated that PL contains high concentrations of chemokines (CCL5/RANTES and CXCL1/2/3) and, in particular, very high concentrations of soluble adhesion molecules (sCD40L, sVCAM and sICAM). Also, a variety of other cytokines, including G-CSF, GM-CSF, IL-la, IL-7, IL-8, MIP-lα, MIP-lβ, TGF-β1 and IFN-γ, are present at concentrations > 10 pg/mL, while bFGF is present at a concentration of 500 ng/mL and TNF-α at 8 ng/mL. We have demonstrated that PDGF-AB/BB, TGF-β1 and bFGF are essential stimuli for the proliferation of MSC. Their inhibition by neutralizing antibodies reduces the MSC growth-stimulating effect of PL by about 75%. Neutralizing antibodies against PDGF-AA did not inhibit PL-stimulated proliferation. This is in line with the expression of PDGF receptor-β (CD 140b) but not PDGF receptor-α (CD 140a) on MSC (43) and the observation that only PDGF receptor-β inhibition suppresses proliferation of MSC (44). The strongest effect of cytokine inhibition by neutralizing antibodies was seen when bFGF was inhibited either alone or in combination with PDGF-BB. Others have even reported inhibitory effects of TGF-β1 on proliferation of inflammatory cells, such as T cells, B cells, dendritic cells, mast cells and eosinophils (45). If this effect was also applicable to our system, one would expect improved proliferation by inhibition of TGF-β1. However, neutralization of TGF-β1 reduced proliferation, albeit the effect of TGF-β1 inhibition was less pronounced compared with bFGF and PDGF-BB inhibition.

The three factors bFGF, PDGF-BB and TGF-β1 seem to be necessary for optimal proliferation of MSC; however, these three factors are not sufficient on their own. A cocktail containing recombinant PDGF-BB, TGF-β1 and bFGF added to a basal medium at concentrations equivalent to the amount present in PL did not stimulate MSC proliferation, even when using cell culture multiwall plates coated with human fibronectin and human placental collagen I—III (see Supplementary Figure 4). Of note, the neutralizing antibodies were not able to abolish the growth-promoting effect of PL completely. Therefore we hypothesize that, in addition to the essential components PDGF-BB, TGF-β1 and bFGF, other constituents of PL are important for its full biologic activity. While our read-out focused on the growth-promoting effects of PL, the various constituents might also be important for differentiation (33), their cytokine resecretion potential (46–48) or migration of MSC (49,50). MSC express receptors for CCL5/RANTES, which has an effect on MSC migration (50,51). A combination of bFGF and CXCL5 seems to be important for invasion/ migration of MSC into 3-dimensional collagen gels (49). TNF-α can up-regulate ICAM-1 expression of MSC and enhance the cells’ migration ability (52), and it can modify characteristics of MSC to improve their engraftment in experimental myocardial infarction (53).

ICAM-1 plays an important role in both innate and adaptive immune responses. It is involved in the transendothelial migration of leukocytes to sites of inflammation, as well as in inflammatory processes and in the T-cell mediated host defense system (54). There, it seems to act as a co-stimulatory molecule activating major histocompatibility complex (MHC) class II-restricted T cells on antigen-presenting cells, and by activating cytotoxic T cells in association with MHC class I on other cell types (55). Interestingly, PDGF-BB has been shown to up-regulate ICAM-1 expression of BM-derived MSC (56). Further, TNF-α, IL-1β and IFN-γ induce sICAM-1 to be shed off from the cell surface of various primary cells and cell lines (54).

MSC bind to endothelial cells in a P-selectin-dependent manner (57). Rolling MSC then interact with very late antigen-4 (VLA-4)/VCAM. This is important for firm adhesion on the endothelial cells (57,58). After integrin β1 blockade, the number of MSC that can be detected in infracted myocardium is reduced (59). Whether the high amount of soluble VCAM that is present in the culture medium might interfere with this process needs to be studied. Thus the complex combination of various cytokines, chemokines and soluble ligands might influence biologic properties, in particular trafficking and homing after *in vivo* administration.

The bioactive components of human- or animal-derived serum are manifold and may therefore impact MSC biology in an unpredictable manner. Thus potential complications that may arise as a result of cell culture conditions and the serum source utilized are a further caveat requiring some consideration.

Whereas the immune phenotype of MSC cultivated in animal-derived serum has been subject of intense investigation, data regarding the use of human sera are still sparse. Recent work comparing serum sources shows that the use of platelet-rich plasma (PRP) results in a significantly higher expansion rate compared with fetal calf serum (FCS)-containing medium. However, a significantly increased secretion of stromal cell-derived factor (SDF)-lα was detected in the supernatant of MSC cultured with FCS, consistent with an enhanced migratory capacity as well as an increased ability to attract CD34^+^ progenitor cells *in vitro* (60). Moreover, MSC cultivated in media supplemented with human AB serum, thrombin-ac-tivated PRP (61) or PL (62) exhibited an enhanced proliferative ability without compromising their differentiation capacity or the immune phenotype. To this end, it could also be shown that PL-cultured MSC have immunomodulatory capacities comparable with their FCS-cultured counterparts, including a beneficial inhibitory effect on immune cell proliferation and an unaffected viral T-cell immunity (63). However, whether these *in vitro* observed differences are of clinical relevance remains to be elucidated.

As soluble factors and cell-surface proteins will provide information to MSC about the local environment, different media supplements and their composition may potentially influence the immunomodulatory properties of MSC. For instance, one of the major cytokines released at the site of inflammation is TNF-α, which has been shown to activate MSC (64), up-regulate surface expression of CD106 (65), stimulate the production of hepatocyte growth factor (HGF) by MSC (66) and enhance expression of the chemokine receptor CXCR4, and therefore facilitates the chemotactic invasiveness of MSC towards SDF-la (67). IFN-γ is known to enhance the immu-nosuppressive behavior of MSC by up-regulation of inhibitory molecule B7-H1 (68) and by inducing indoleamine-pyrrole 2,3-dioxygenase (IDO) production (69). Like IFN-γ, IL-1β appears to be one of the cytokines that can prime MSC *in vitro.* Interestingly, IL-1β alone may be sufficient to prime MSC, whereas the effect of IFN-γ may be amplified in the presence of other pro-inflammatory cytokines, such as IL-ip and TNF-α (70). Among the factors MSC produce to suppress immune reactions, prostaglandin E2 (PGE2) is one of the most studied molecules and has been shown to be secreted by MSC in response to IL-6, IFN-γ and TNF-α (64). Also, CCL2 and IL-8 have a positive impact on MSC survival, proliferation and adhesiveness to acute lymphoblastic leukemia (ALL) cells (71). While there is now an increasing amount of data illustrating the effects of MSC-secreted factors on other cell types, information regarding the effect of the milieu on MSC biology is still sparse. Further experimental work identifying important components of PL beyond those presented here and delineating their individual and synergistic effects on MSC biology should allow the preparation of tailored fractions of PL enriched for important components, or cocktails composed of recombinant factors with equivalent activity. In conclusion, it is of paramount importance to understand and investigate the impact of supplemental factors on MSC biology, generating final cell-culture products with specific custom-designed, application-oriented properties.

## Supplementary material available online

Supplementary Figures 1–4 to be found online at http://www.informahealthcare.com/cyt/doi/10.3109/14653249.2012.655420
