# Supplementary material for: Platelet lysate from whole blood-derived pooled platelet concentrates and apheresis-derived platelet concentrates for the isolation and expansion of human bone marrow mesenchymal stromal cells: production process, content and identification of active components
Source: Cytotherapy. 2012 May;14(5):540–54. doi: 10.3109/14653249.2012.655420 (PMC3400099; doi:10.3109/14653249.2012.655420)
Supplement: Supplementary file 1 [file mcyt14-540-SD1.pdf]

Supplementary material for Fekete N *et al.* Platelet lysate from whole blood-derived pooled platelet concentrates and apheresis-derived platelet concentrates for the isolation and expansion of human bone marrow mesenchymal stromal cells: production process, content and identification of active components, *Cytotherapy*, 2012;14:540–554.

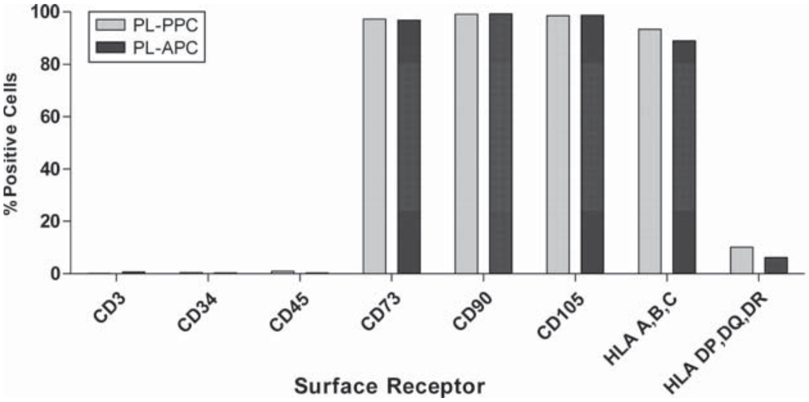

% Positive cells

	PL-PPC	PL-APC
CD3	0.11	0.62
CD34	0.34	0.28
CD45	0.91	0.29
CD73	97.22	96.87
CD90	99.13	99.30
CD105	98.53	98.80
HLA A,B,C	93.43	88.91
HLA DP,DQ,DR	10.08	6.24

Supplementary Figure 1. Flow cytometry analysis of large-scale expanded MSC using a single-step isolation and expansion protocol. The cells were cultivated in medium supplemented with either 10% PL-PPC (white bars) or PL-APC (black bars).

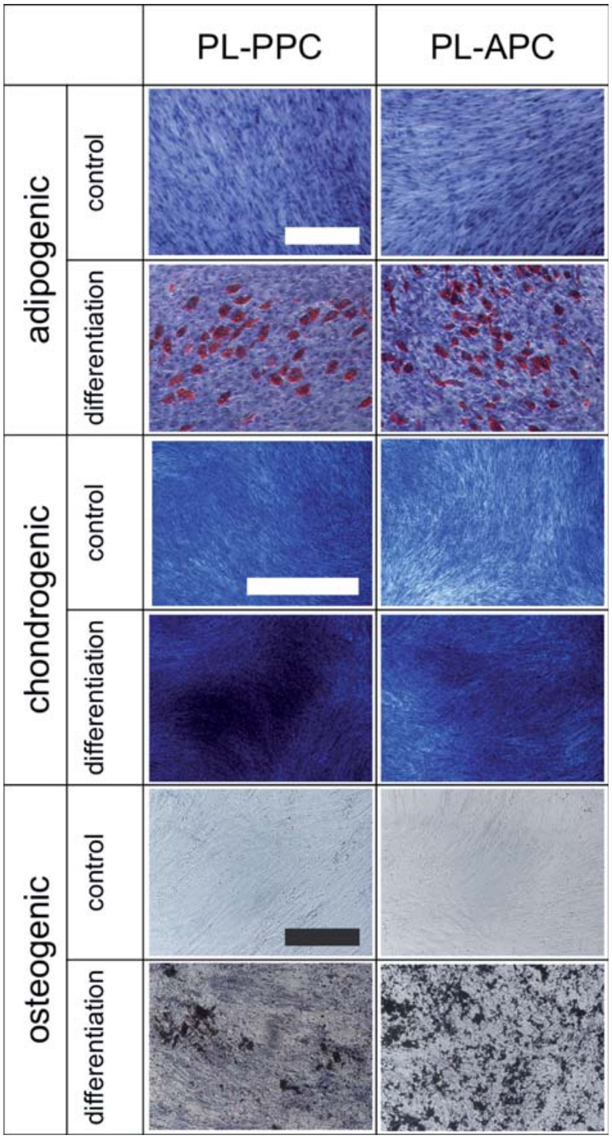

Supplementary Figure 2. Differentiation potential of large-scale expanded MSC.Large-scale expanded MSC grown in medium containing 10% PL-PPC or PL-APC were tested for their capacity of adipogenic, chondrogenic and osteogenic differentiation. Control: MSC grown in medium without differentiating factors, differentiation: MSC grown in differentiating medium. Bars indicate 250 µm for adipogenic and osteogenic, 1000 µm for chondrogenic differentiation, respectively.

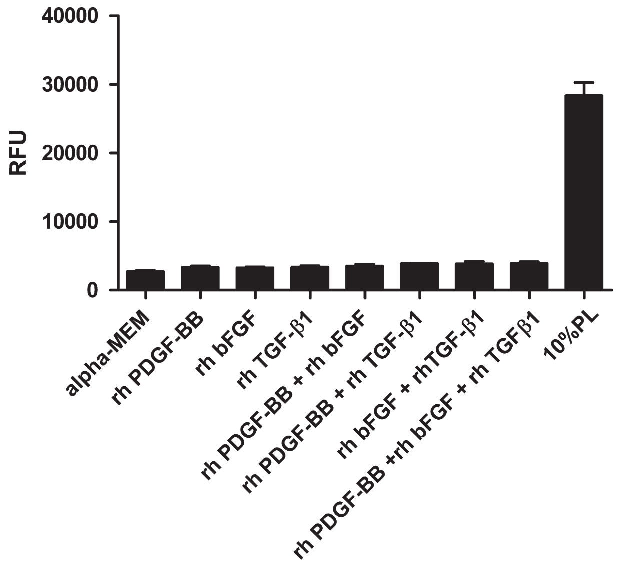

Supplementary Figure 3. MSC proliferation was stimulated by recombinant human PDGF-BB, bFGF and TGF-?1. Cells were cultivated in medium supplemented with the indicated cytokines or 10% PL-PPC. Results are expressed as mean and SD values of one representative experiment, which was repeated once (RFU = relative fluorescence units; rh = recombinant human).

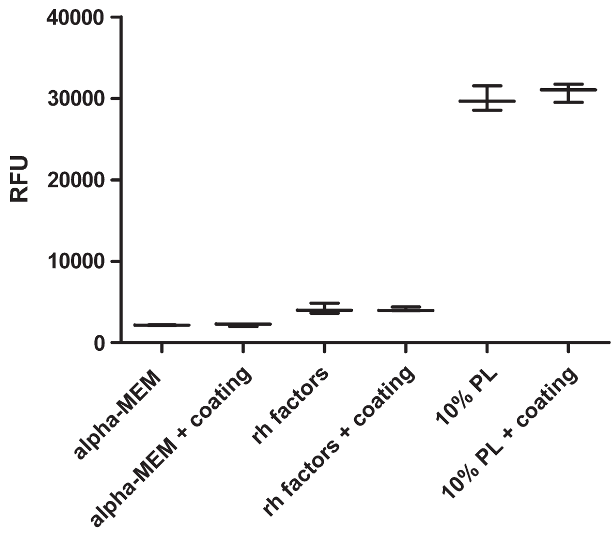

Supplementary Figure 4. Influence of coating of cell culture surface with human fibronectin and human collagen I-III on MSC proliferation. After coating the cell culture surface, MSC were cultivated in medium supplemented with either a combination of recombinant human PDGF-BB, bFGF, Activin A, IGF-I and Dexamethasone (rh factors) or 10% PL. Results are expressed as median values in a Tukey's Box-Whisker-Plot of a representative experiment (RFU = relative fluorescence units; rh = recombinant human).
